# Chemically Modified Pineapple Leaf Fibre as a Filler of Polyurethane-Based Composites

**DOI:** 10.3390/ma18020386

**Published:** 2025-01-16

**Authors:** Piotr Szatkowski, Rafał Twaróg, Karolina Sowińska, Kinga Pielichowska

**Affiliations:** Department of Glass Technology and Amorphous Coatings, Faculty of Materials Science and Ceramics, AGH University of Krakow, Al. Mickiewicza 30, 30-059 Krakow, Poland; pszatko@agh.edu.pl (P.S.); rtwarog@agh.edu.pl (R.T.); sowinska@student.agh.edu.pl (K.S.)

**Keywords:** pineapple fibres, biocomposite, polyurethane

## Abstract

Pineapple leaf fibres represent a biodegradable raw material sourced from renewable resources whose use contributes to reducing the carbon footprint and limiting the amount of waste generated. Their potential applications can effectively decrease the industry’s dependence on plastics and support sustainable development, which should accompany the production of modern materials. In this study, polyurethane-based composites reinforced with various types of natural cellulose fillers were developed and investigated. Microcrystalline cellulose and unmodified and chemically modified pineapple leaf fibres were used as reinforcements. The mechanical and thermal properties of the produced materials were determined and compared. The results of the tests indicated that both microcrystalline cellulose and pineapple leaf fibres contributed to a reduction in the mechanical properties of polyurethane. A varying impact of fillers on the Young’s modulus of the biocomposites was observed. The presence of natural modifiers influenced an increase in the melting temperature of the composite compared to the pure polyurethane. Integration of natural pineapple fibres into composite represents a step toward a more sustainable future, combining economic benefits with environmental care. The mechanical characteristics of composite materials were enhanced by modified fibres, compared to their unmodified counterparts. This improvement comes from the unique structural properties of the modified fibres. When polyurethane (PU) is used as the matrix material, it effectively fills the interfibrillar voids, creating a more cohesive bond between the components.

## 1. Introduction

Plant fibres obtained from various parts of plants have been used for centuries. Initially used mainly in textile production, they now find increasingly broader applications in industry. Their unique properties, such as strength, flexibility, lightness, and biodegradability, make them an attractive raw material for many industrial sectors [1,2,3,4]. The diversity of plant fibres is enormous. Each type of fibre, whether derived from seeds (cotton), stems (flax, hemp), leaves (sisal), or fruits (coconut), has specific characteristics that predispose it to certain applications [1,2,5,6]. One of the strongest plant fibres is that obtained from pineapple leaves. Pineapples are cultivated for their edible fruits, while leaves constitute waste material. The scale of by-product waste in the form of leaves is very large—approximately 250 metric tons of wet plant residues per hectare—because pineapple is the most common tropical fruit, after bananas and citrus fruits. Many producers currently lack effective waste management systems, often resorting to practices that lead to environmental degradation, such as burning or simply discarding waste. This not only contributes to greenhouse gas emissions, but also poses risks to soil health and pest management. Therefore, innovative approaches to valorise pineapple waste could mitigate these environmental impacts while improving the economic viability of pineapple farming [7,8,9,10,11,12]. In connection with the search for new natural materials as alternatives to synthetic materials, interest has turned to pineapple leaves as a source of natural fibres. This simultaneously leads to minimising the waste of renewable resources. Pineapple leaf fibres are mainly obtained through mechanical methods. Fresh leaves contain an average of 2–3% fibres. Pineapple leaf fibre is a multicellular lignocellulosic fibre composed of polysaccharides, large amounts of lignin, and other chemical substances such as fats, waxes, pectins, pigments, etc. These are medium-length white fibres, with high smoothness, a silk-like glossy silky surface, and high tensile strength. They have a very soft surface compared to other natural fibres, making them good at absorbing dyes and retaining colour. They are also characterised by high specific strength, stiffness, resistance to bending and twisting, and hydrophilicity due to their high cellulose content (70–82%). Table 1 shows the properties of selected types of plant fibres. It can be seen that pineapple leaf fibres perform best in terms of tensile strength and Young’s modulus (sometimes over seven-fold difference, e.g., for cotton) compared to other types of fibres. At the same time, they have the highest density, about 25% higher than sisal or cotton, and the lowest percentage of elongation at break, with a value almost nine times lower than cotton [13,14,15,16,17].

Cellulose, the main component of PALF, exists in two distinct forms: crystalline and amorphous. In the crystalline phase, there are bundles of microfibrils, composed of (1–4) β-D-glucan chain assemblies, which are strongly connected by hydrogen bonds [18]. The amorphous phase consists of randomly arranged cellulose and hemicellulose which minimally contribute to the structural and mechanical rigidity of the fibre [19,20,21].

Research has shown that chemical treatment of natural fibres can not only improve their surface morphology but also enhance the mechanical properties of the fibre. The treatment of natural fibres is related to their hydrophilic nature, which discourages good fibre-matrix bonding with most hydrophobic matrices [22,23,24,25,26]. For this reason, researchers in the field of biological fibres have investigated various chemical treatment techniques for pineapple fibre, not only to improve the fibre–matrix bonding capability for better composite reinforcement applications but also due to changes in the structure of pineapple fibres [27,28,29].

Research on composites with thermoplastic matrices modified with natural fibres is well documented in literature. Studies mainly focus on composites with cellulose fibres derived from flax, cotton, and hemp, due to the length of cellulose fibres and their availability [30,31,32]. Research on PALF/polyurethane composites has innovative potential, due to the use of renewable raw materials, the possibility of obtaining unique material properties, and optimization of manufacturing processes. Comparison with studies on other natural fibre composites allows for the assessment of competitiveness and the identification of specific advantages of PALF. The use of PALF instead of synthetic fibres (e.g., glass fibre, carbon fibre) aligns with the trend of sustainable development and circular economy. Pineapple is an agricultural crop and its leaves are a waste product after fruit harvesting. Utilising them as a composite reinforcement reduces waste and promotes the use of renewable resources. This, in itself, is innovative in the context of seeking ecological alternatives to traditional materials.

The influence of natural plant-based fibres has also been investigated in the case of polyurethane matrix [30,33,34,35,36]. Natural fibres contain hydroxyl groups that can interact with the polymer matrix, potentially leading to weak interfaces if not properly treated. Chemical modifications are often necessary to improve compatibility and bonding strength between the hydrophilic fibres and hydrophobic polyurethane matrices [36,37]. Using natural fibres in polyurethane composites offers advantages such as lower cost and biodegradability compared to synthetic alternatives. This aligns with growing environmental concerns and the push towards sustainable materials in various industries. Natural fibres significantly enhance the mechanical properties of polyurethane composites, while also introducing challenges related to moisture absorption and interfacial bonding that can be addressed through chemical treatments and modifications [37]. Pineapple leaf fibres are characterized by high mechanical strength (especially tensile strength), low density, and good stiffness. Combining these properties with an elastic polyurethane matrix can lead to the acquisition of materials with unique properties, such as high strength, while maintaining elasticity and impact resistance. To date, there have been no studies on the impact of modified pineapple leaf fibres on polyurethane matrix.

In this article, the authors attempt to thoroughly investigate the impact of polyurethane matrix modification with pineapple fibre and chemically modified pineapple fibre. Furthermore, this paper provides information on how PALF treatment affects the mechanical properties of the fibre.

## 2. Materials and Methods

### 2.1. Materials Preparations

#### 2.1.1. Materials for the Polyurethane Synthesis

The composite matrix was composed of polyurethane. Its synthesis involved three components: 4,4′-methylenediphenyl isocyanate (MDI) (Sigma Aldrich, Darmstadt, Germany), polytetrahydrofuran (PTHF) dried for 12 h at 60 °C in a low vacuum, with an average molar mass of 2000 (Sigma Aldrich, Germany), and butane-1,4-diol (BDO) (Sigma Aldrich, Germany) as a chain extender. All reagents were used as received.

#### 2.1.2. Preparation and Chemical Modification of Pineapple Leaf Fibres

**Figure 1:**
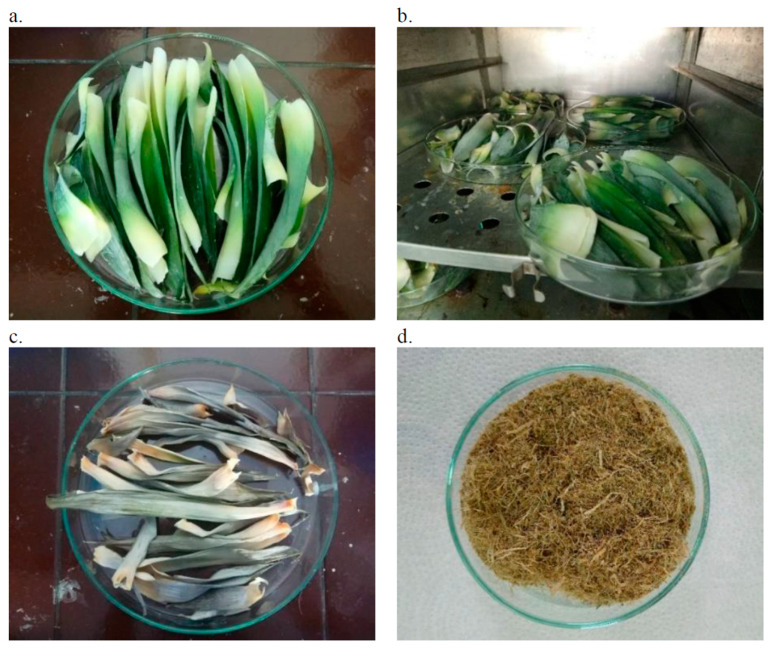
Process of obtaining fibres from pineapple leaves. Preparation of fresh pineapple crown leaves (**a**); leaf drying (**b**); leaves after the drying process (**c**); leaves after grinding in an impact mill (**d**).

The leaves of the pineapple crown were spread on glass Petri dishes (Figure 1a), which were subsequently placed in a dryer (Figure 1b) for a duration of 72 h at a temperature of 85 °C, ensuring that the water did not reach the boiling point, to prevent potential damage to the fibres. The dried leaves (Figure 1c) were then ground using a hammer mill (see Figure 1d), resulting in a filler in the form of fibres. The fibres were divided into two portions: one was utilised in its unmodified state for further composite formation, while the other underwent chemical modification.

**Figure 2:**
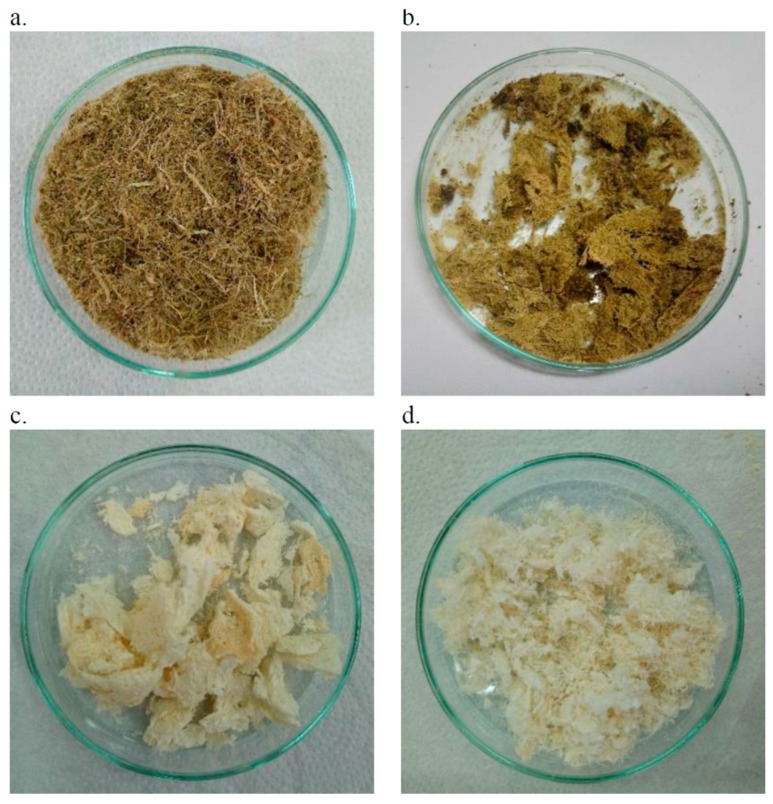
Stages of pineapple leaf fibre modification. Fibers after milling process (**a**); fibres after chemical treatment in NaOH and Na_2_SO_3_ solution, washing and draining (**b**) fibres after chemical treatment in H_2_O_2_ solution, washing and drying (**c**) final obtained fibres ground in impact mill (**d**).

Chemical modification was based on the use of three components. Mercerization (involving NaOH and Na_2_SO_3_) allows for the dissolution of polysaccharides and the saponification of waxes and oils adhering to the fibre surface. The second stage, bleaching (using H_2_O_2_), aims to remove dyes, lignin, and other non-cellulosic substances present in the fibres. A solution of 4.5 M NaOH and 0.4 M Na_2_SO_3_ was prepared in 100 cm^3^ of distilled water within a 250 cm^3^ flask, into which the previously prepared fibres were immersed. A boiling setup—comprising a stand, condenser, and heating mantle—was arranged and the flask was placed within it to conduct the boiling process for 3 h. After this period, the fibres were drained on a filter, rinsed thoroughly with distilled water, and the draining process was repeated. The prepared fibres were then transferred to a glass Petri dish and left to air dry for 24 h (Figure 2b). After drying, the fibres were transferred back to the flask and submerged in a 30% H_2_O_2_ solution. After approximately 15 min, the fibres were again drained on a filter, and this process was repeated. The fibres were rinsed with a large volume of distilled water, drained once more, and placed on a dish. The dish was transferred to a dryer set at 100 °C and left to dry for 24 h (Figure 2c). Upon drying, the fibres were ground again using a hammer mill (Figure 2d). As a result of the chemical modification, there was a significant reduction in fibre mass, with a seven-fold decrease observed, attributed to the removal of non-cellulosic substances (including dyes) and resulting in a colour change of the fibres from green to pale yellow. After the chemical modification process, the fibre residue was 65% by weight ±3%.

#### 2.1.3. Composites Preparation

Three types of modifiers were used for the composites preparation: microcrystalline cellulose (MCC), unmodified pineapple crown leaf fibres (PALF), and chemically modified fibres (modPALF). MCC was chosen as it is the purest form of cellulose and PU/MCC composites referred to PU/PALF and PU/modPALF composites. A polyurethane (PU) matrix containing 80% of the soft segments was obtained. For the synthesis of the composites, 4 g PTHF was first weighed into a reaction vessel. The vessel was sealed and transferred to a dryer at 60 °C. A needle-fitted syringe was placed in a glass beaker. 0.875 g of MDI was then inserted into the syringe. The syringe was subsequently placed in the dryer at 60 °C for 5 min until the MDI melted. Next, glass Petri dishes were prepared by lining them with aluminium foil, smoothing their surfaces, and placing them in the dryer at 60 °C until completely dry and heated (approximately 30 min). The filler was weighed in the reaction vessel containing the polyol and mixed using an ultrasonic stirrer for 3 min. The vessel was resealed and returned to the dryer for an additional 15 min. Once the polyol and isocyanate had melted, the actual synthesis commenced. A suitable amount (0.132 g) of BDO was added to the reaction vessel containing the polyol and filler. The prepared mould and MDI were removed from the dryer and injected into the reaction vessel, stirring vigorously. After thorough mixing, the resulting mixture was poured into the mould and placed in a dryer set at 80 °C for approximately 24 h. A summary of the composites obtained is shown in Table 2.

**Figure 3:**
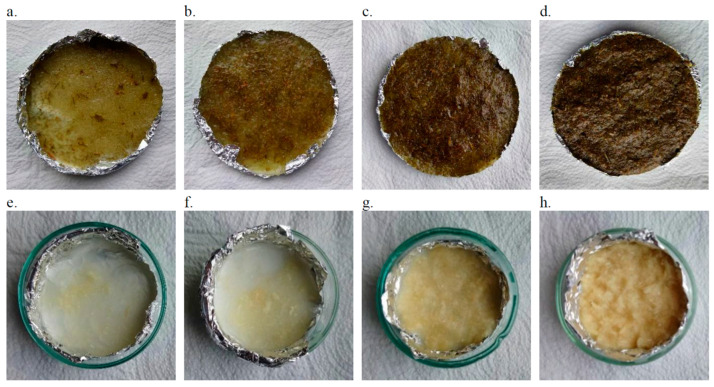
Polyurethane composites modified with PALF (**a**–**d**) and modPALF (**e**–**h**) with filler content: 1% (**a**,**e**), 2% (**b**,**f**), 5% (**c**,**g**), 10% (**d**,**h**).

Selected samples are shown in Figure 3.

### 2.2. Methods

The mechanical strength characteristics of the samples were determined through static tensile testing in accordance with the ISO 527 standards [38]. The tests were carried out using a Zwick/Roell 1445 RetroLine testing machine (Ulm, Germany), series no.: 736.695. Standard dimensions for the samples were used, with a thickness of 4.0 ± 0.2 mm, a measurement section width of 10 ± 0.2 mm and a total length exceeding 150 mm. The test speed for the modulus was set at 1 mm/min, while the testing speed for the tensile properties was maintained at 2 mm/min. The results yielded a force–deformation curve, along with parameters such as tensile strength, tensile modulus, maximum force, and the corresponding deformation at that force.

Thermal properties were investigated using a differential scanning calorimeter (DSC 1) from Mettler Toledo (Greifensee, Switzerland). A sample weighing approximately 5.5 mg was cut from each material, sealed in pierced aluminium pans, and placed in the device. STAR Thermal Analysis Software (ver. 1.6) was employed for controlling the apparatus and processing the results. The materials were heated, cooled, and reheated over a temperature range from −30 °C to +250 °C, with a heating and cooling rate of 10 °C/min.

The thermogravimetric analysis (TG) was performed using a TGA550 Discovery (TA Instruments, New Castle, DE, USA) thermal analyser. The samples, weighing ca. 10 mg, were heated over a temperature range from 50 °C to 600 °C at a rate of 10 °C/min in a nitrogen atmosphere. The TG curves were analysed to determine the temperatures at which mass losses of 1%, 2%, 3%, 5%, and 50% occurred (T_1%_, T_2%_, T_3%_, T_5%_, T_50%_).

Microscopic observations of plant additives and produced biocomposites were carried out using a NOVA NANO SEM 200 (Thermo Fisher: Waltham, MA, USA) scanning electron microscope. Samples were coated with carbon before SEM observations.

## 3. Results and Discussion

### 3.1. Tensile Strength Test

The mechanical properties of the produced materials were determined through static tensile testing. For each of the tested samples, the following parameters were established: R_m_—tensile strength [MPa], E—Young’s modulus [MPa], and ε_max_—elongation at break [mm]. Detailed results are compiled in Table 3.

In accordance with expectations, pure PU exhibited the highest tensile strength. The composite MCC/PU, with a 1% of filler, recorded a tensile strength value that was over 20% lower than that of pure PU. For all composites examined, a decrease in tensile strength was observed as the filler content increased. This reduction in strength may be attributed to the presence of substances that impart rigidity to fibres, such as crystalline cellulose. Exceptions were noted for both PALF/PU and modPALF/PU composites, where the tensile strength values for the 1% reinforcement were lower than those for the 2% reinforcement. This relationship suggests that, at very low fibre content (1%), the fibres act as inclusions or defects within the matrix, and only after surpassing a certain threshold (2%) do we observe an enhancement in material strength due to reinforcement. When comparing the results of composite materials, significantly lower values were observed for PALF/PU composites. This could be due to the presence of substances within the fibres, such as lignin, that contributes to fibre rigidity, as well as hemicellulose and pectins that diminish their mechanical properties. The tensile strength results for modPALF/PU composites were higher than those for composites containing unmodified fibres, indicating that the chemical treatment of the fibres effectively improved their properties, as anticipated.

In the literature, an increase in mechanical bending strength (of PU foam) of up to 40% was observed with 1% agave fibre content [39]. Similarly, in the case of PU foams, the influence of coconut fibres (5%) was observed to improve the mechanical properties of polyurethane composite; however, a decrease in the flexural modulus was observed compared to PU foams without reinforcement at higher fibre content, in the range of 10–20% by weight [40]. A similar trend was observed in PU foams reinforced with hemp fibres [41]. However, these studies concern foams and not non-porous polyurethanes. In tensile and tear tests, pineapple fibres introduce defects, and a decrease in tensile strength values is observed.

**Figure 4:**
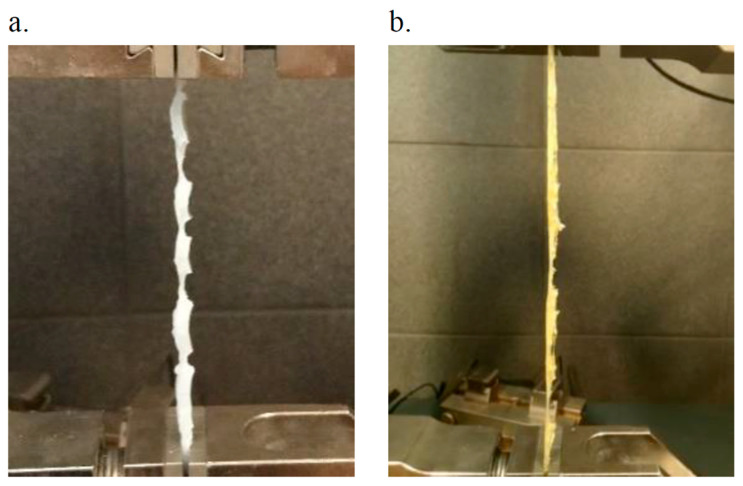
Visible bridging of the specimen during static tension test (**a**) modPAFL/PU (**b**) PALF/PU.

The Young’s modulus for pure PU was approximately 140 MPa. In the case of the composites, only the 1% weight fraction of the PALF filler resulted in a lower modulus value compared to that of pure PU, while the remaining composites exhibited significantly higher values. The composite with 10% modPALF/PU displayed the highest modulus, achieving a value of 710 MPa, which is five times greater than that of pure PU. A clear relationship is evident between the fibre content in the composite and Young’s modulus. The value of Young’s modulus depends on the intended application of the final material. This implies that a material with a high Young’s modulus is not always desirable and vice versa. For instance, if there is a need to reduce strength properties while maintaining the modulus at a certain level, PALF can be used as a modifier. Regarding the parameter of elongation at break, all composites exhibited lower values compared to unmodified PU. The highest values among the composites were obtained for the samples with 1% and 2% PALF/PU. On the contrary, the lowest elongation at break was observed in the sample with 10% modPALF/PU, which also had the highest Young’s modulus, measuring 6.36%. The favourable achievement of similar elongation values for pineapple fibre-modified samples compared to pure PU may be influenced by the observed bridging stage of the sample (Figure 4). The fibres acting as modifiers impeded crack propagation, thereby enhancing the performance of the material.

Synthesis in situ of PU in the presence of filler allows for the formation of a chemical bond by incorporating cellulose chains into the PU chains as crosslinker of hard segments. Due to the increased content of hard segments, an improvement in the properties of the matrix material, such as Young’s modulus, hardness, and maximum service temperature, is anticipated. In contrast, pure PU, characterized by a higher content of soft segments, is expected to exhibit superior flexibility, elongation, and resistance to low temperatures. These assumptions are related to theoretical considerations. In the case of composites with randomly distributed fibres, predicting mechanical properties becomes challenging, especially when the testing typically focusses on a single direction. Additionally, numerous practical factors can disrupt certain properties, including synthesis or modification conditions, mechanical damage to fibres during their preparation, or the limited experience of the individual conducting the process. It is also noteworthy that efforts have been made to produce solid materials; however, porous materials were obtained instead. The pores within the material may have exposed the fibres, subjecting them directly to stresses and factors that should ideally remain in contact with the matrix [42].

### 3.2. Investigation of Material Phase Transitions Under the Influence of Heat Flow (DSC)

#### 3.2.1. Results Obtained for MCC/PU Composites

In the course of the study, each material was initially heated, subsequently cooled and then reheated. This method of investigation allows for the acquisition of additional information, including less apparent differences between the materials studied, while also eliminating the thermal history, which includes the effects connected to preparation, processing, and storage of the material. For each curve related to the first heating phase, tables were developed that contain information regarding the phase transitions associated with the melting of soft segments. In addition to the melting of soft segments, two additional temperature ranges (60–110 °C and 180–220 °C) can be distinguished for each material, corresponding to more or less noticeable changes related to the transitions of PU hard segments.

**Figure 5:**
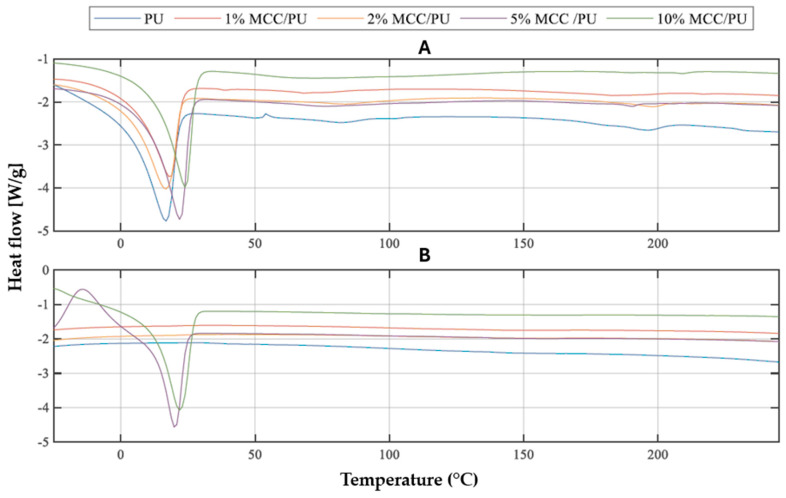
DSC curves from the first (**A**) and second (**B**) heating run of PU and PU/MCC composites.

Table 4 presents information regarding the initial phase transitions observed in the curves corresponding to the first heating run (Figure 5A). It was observed that, as the filler content increases, there is a corresponding rise in the melting temperature, while cold crystallization was found in the composite with a 5% MCC content during the second heating run. For these two materials, melting of the resulting crystalline phases was observed during the second heating. These findings suggest that cellulose additives enhance the nucleation and crystallization processes of soft segments.

#### 3.2.2. Results Obtained for PALF/PU Composites

**Figure 6:**
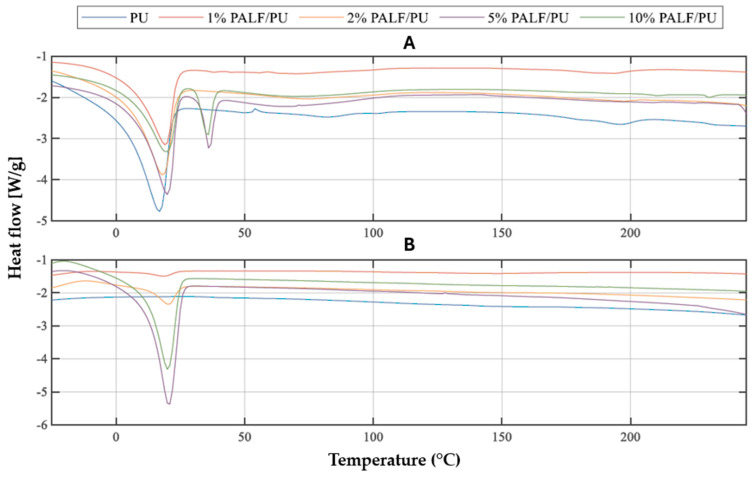
DSC curves for the first (**A**) and second (**B**) heating run for PU and PU/PALF composites.

In the case of the curves representing the results of testing PALF/PU composites (Figure 6), unlike those for PU or MCC/PU composites, a second melting was observed for certain mass fractions of the filler. For low PALF contents (1% and 2%), single transitions were observed, with temperatures higher than those of pure PU and lower heat of fusion (Table 5). For PALF/PU composites with 5% and 10% filler content, two phase transitions were observed, with the first characterized by much higher heat of phase transition. The presence of a second endothermic peak at ca. 30–40 °C may indicate melting of the PTHF fraction. In such large amounts of soft segments, some part of the soft segments can undergo strong phase separation and behave as pure PTHF. Moreover, as this effect was observed for samples with the highest content of PALF, it suggests that the presence of PALF can hinder the mobility of macromolecules and complete polymerization reaction, and in consequence, melting of unreacted PTHF can be observed. In the second heating cycle, only one melting transition is observed for each of the PALF/PU composites. That suggests that the composites with such fillers should be kept at an elevated temperature, higher than that of the standard procedure (80 °C for 24 h), to complete the polymerization reaction. It should also be noted that this effect was not observed for the PU/PCC composites. The temperatures corresponding to the end of the melting process are very similar, at around 20 °C. The heat of phase transition for the first two samples (1% and 2% PALF/PU) are low (up to 3 J/g), whereas for composites with higher filler content, these values reach approximately 34 J/g. This suggests that the PALF fibres can act as a nucleant for the soft segment crystallization process.

#### 3.2.3. Results Obtained for modPALF/PU Composites

**Figure 7:**
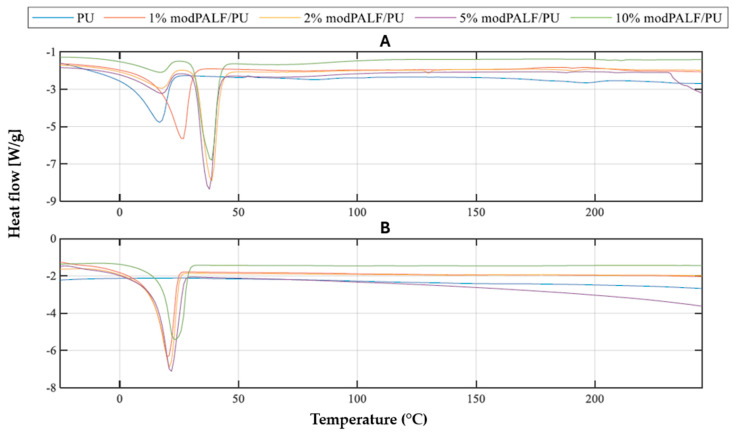
DSC curves for the first (**A**) and second (**B**) heating run of PU and PU/modPALF composites.

In the case of modPALF/PU composites (Figure 7) with a filler content higher than 1%, two transitions can be observed during the first heating, unlike the PALF/PU composites where the first transition occurs at a lower temperature (Table 6). The first transition can be connected to the melting process of soft segments, while the second peak can be connected to the melting of PTHF. This can be explained in the same way as for the PU/PALF composites and suggests that another temperature treatment after preparation should be applied. In the second heating cycle, as with other composites, single melting transitions are observed. These occur at temperatures within the range typical for melting of PTHF soft segments.

By comparing the values obtained for all studied materials, it can be concluded that all types of modifiers have a noticeable impact on the PU matrix. The modPALF/PU composites exhibited the highest resistance to temperature-induced changes, with the phase transition temperatures, the temperature range of phase transitions, and heat of phase transitions. The PALF/PU composites demonstrated similar trends and properties, whereas the least pronounced effects were observed in the MCC/PU composites. It was also noted that there is a correlation between the proportion of the modifier and the delay in transitions occurring within the material—an increased filler content increases the melting temperature of the material.

### 3.3. Thermal Stability Testing of Composites

**Figure 8:**
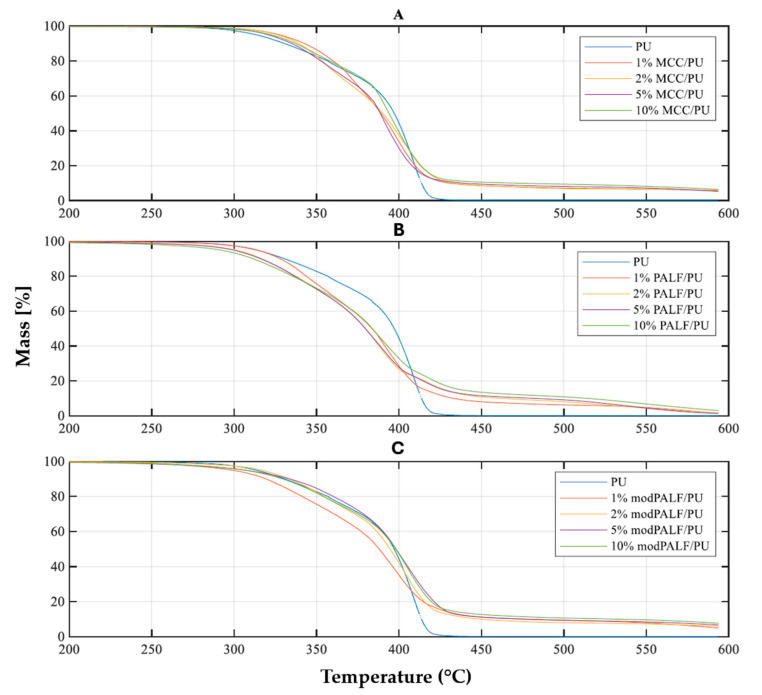
TG curves of polyurethane and its composites with MCC (**A**), PALF (**B**) and modPALF (**C**).

**Figure 9:**
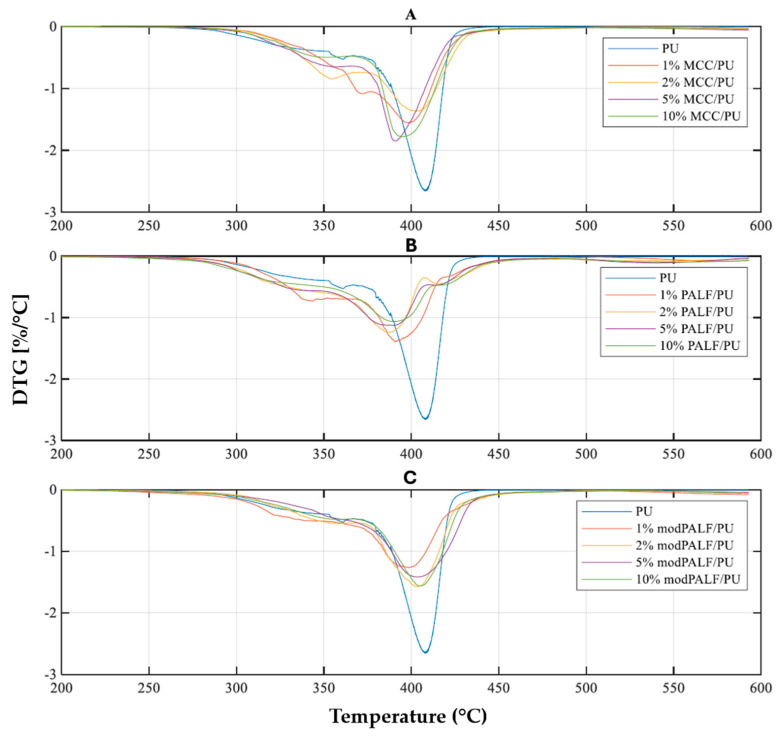
DTG curves of PU and its composites modified with MCC (**A**), PALF (**B**) and modPALF (**C**).

Results of thermogravimetric measurements are presented in Figure 8, while DTG curves are presented in Figure 9.

Based on the TG curves it was estimated that, at a final temperature close to 600 °C, the remaining mass of polyurethane (PU) is nearly 0%, indicating that the material has degraded almost completely. In the case of the composites, the residue varies from 2% to 7%, depending on the filler used. This suggests that a thermally stable residue remains in the material. For each of the studied materials, temperatures corresponding to mass loss of 1%, 2%, 3%, 5%, and 50% were also determined (T_1%_, T_2%_, T_3%_, T_5%_, T_50%_). The results are presented in Table 7.

In the case of polymers, the thermal stability can be assumed as the temperature at which a 1% or 2% mass loss occurs. For pure PU, the onset of degradation occurs at 280 °C. The addition of microcrystalline cellulose (1% MCC/PU) increased the thermal stability of the PU matrix by 12 °C, while for composites modified with modPALF (1% modPALF/PU), the thermal stability was 31 °C lower. For both PALF and modPALF, the thermal stability of the PU matrix was lower compared to unmodified PU and PU/MCC composites. Similar results have been observed by other researchers investigating the effect of cellulose on the polyurethane matrix in a composite [43].

From analysis of the DTG curves (Figure 9), it was observed that degradation occurred in two stages for pure PU and the MCC/PU and modPALF/PU composites, while a three-stage degradation was observed for the PALF/PU composite. The stages may correspond to the cleavage of urethane bonds in hard segments (degradation of hard segments), the breakdown of polyol chains (degradation of soft segments), and the oxidation of the solid residue, or in the case of composites containing unmodified fibres, non-cellulosic substances. Thus, two to three temperature values were determined for each material, corresponding to the greatest mass losses (maximum rates of change: T_maxDTG1_, T_maxDTG2_, T_maxDTG3_). The rates of mass change associated with these temperatures were also identified (maxDTG1, maxDTG2, maxDTG3). Values are displayed in Table 8.

During the first stage of degradation—degradation of hard segments (maxDTG1)—it can be observed that the addition of modPALF ensures a slowdown in the degradation process; however, this process occurs in the lowest temperature range compared to other studied materials. Subsequently, PALF also exhibits a beneficial effect when present with a filler content of 2% or greater. On the other hand, it is characterized by the highest degradation rate, which is more than twice that of pure PU at a filler content. Degradation occurs most rapidly for these composites, although it is delayed compared to the other materials. In the temperature range corresponding to the degradation of soft segments, pure PU exhibited the highest temperature, coinciding with the highest rate of degradation. PALF/PU composites displayed the lowest rates of degradation; however, this degradation occurred in a lower temperature range than for the other composites. Considering both the temperature and rate of process, the most thermally stable materials were found to be the modPALF/PU composites. The third stage (DTG3) observed in PALF/PU composites may result from the presence of certain substances in the fibres that remain after matrix degradation and which themselves degrade at elevated temperatures. The absence of further stages for the other composites is attributed to the lack of similar substances, as they are either not present in MCC or have been removed during the modification process of pineapple fibres.

**Figure 10:**
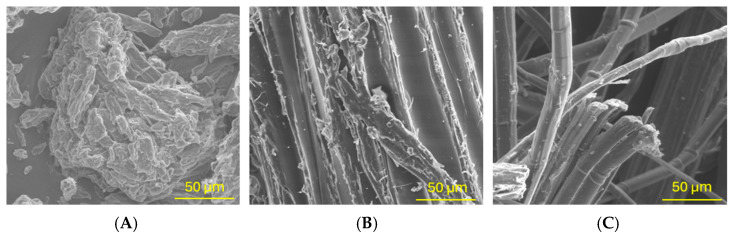
SEM image of biocomposite samples with MCC (**A**), PALF (**B**) and modPALF (**C**).

Figure 10 presents SEM images of biocomposite samples containing various types of modifiers.

SEM studies revealed significant differences in the microstructure of the PU composites. For composites with MCC only, small particles of MCC dispersed in the PU matrix can be seen. For composites with pineapple leaf, fibres dominate the fibrous microstructure. It should also be noted that modPALF fibres exhibit a much lower level of surface defects compared to unmodified pineapple leaf fibres. This effect can be attributed to the chemical modification that leads to the dissolution of polysaccharides and the saponification of waxes and oils adhering to the fibre surface.

## 4. Conclusions

In this study, we successfully obtained PU composites reinforced with pineapple leaf fibres, demonstrating their potential as sustainable materials for various applications. Natural fibres derived from pineapple leaves possess unique properties compared to fillers that have previously been used. The choice of this type of material is aligned with the current trend that is focused on natural resources, highlighting their potential as sustainable materials in diverse applications. In addition to ecological benefits, these materials also offer economic advantages.

The fibres were obtained through the drying and grinding of leaves. Some of the resulting fibres were used directly as a modifier, while the others underwent a chemical modification process. PU composites were obtained by in situ polymerization that allows the incorporation of cellulose chains into the PU backbone, which served as extensions of the hard segments, thereby ensuring improved mechanical properties.

The developed materials were subjected to mechanical and thermal testing. The results of the static tensile tests indicated that the presence of a modifier leads to a reduction in the tensile strength of the PU, regardless of the type of filler used. As the weight fraction of the fibres increases, a decline in tensile strength is observed. For PALF/PU composites, lower values were recorded compared to MCC/PU composites, while modPALF/PU demonstrated a noticeable improvement relative to composites containing unmodified fibres. The presence of hemicellulose, lignin, pectins, and waxes contributes to water retention in the fibres (which is facilitated by the hydrophilic hydroxyl groups), thus conditioning the hydrophilic nature of the fibres, which reduces compatibility with hydrophobic materials. The low compatibility between the filler and the matrix, along with the weak interfacial strength, diminishes the resulting properties of the composite. Modified fibres positively influenced the mechanical properties of the composites compared to unmodified ones, due to their lack of these substances. In such fibres, the interfibrillar spaces are voids, and the PU used as a matrix fills these spaces, ensuring better bonding. The incorporation of fibres for modifying the matrix increased Young’s modulus in nearly all cases, compared to pure PU. An increase in modulus values was observed with an increase in fibre content in the composite. Surface modification of pineapple fibres promotes strengthening, increased strength, and increased elasticity. Conversely, there was a significant decrease in maximum elongation at break, similar to tensile strength, particularly for composites containing 5% and 10% filler. Valuable information was also obtained from thermal analysis. Changes in the melting temperature range of soft segments in PU were observed for PU/PALF and PU/modPALF composites. Additionally, the heats of the phase transition were higher than those for pure PU and MCC/PU composites. The results indicated that the thermal degradation profile depends on the applied modifier: for PALF/PU composites, degradation occurred in three stages, whereas for other materials, it occurred in two stages.

In summary, the research conducted has contributed to a better understanding and assessment of the impact of pineapple fibre modification on the properties of PU composites. This research highlights the promising role of natural fibres in enhancing the performance of polyurethane composites, while simultaneously promoting sustainable development in material innovation. From a future development perspective, it is also worthwhile to consider the use of biodegradable materials for the matrix, such as polylactic acid (PLA) or polyhydroxybutyrate (PHB), to create a fully biodegradable composite. Undoubtedly, the use of natural and renewable additives such as pineapple fibres should positively impact the environment, while minimizing the rapidly increasing amount of industrial waste.

## Figures and Tables

**Table 1:** Properties of selected plant fibres [16].

Plant Fibre	Density [g/cm^3^]	Tensile Strength [MPa]	Young’s Modulus [GPa]	Elongation at Break [%]
Banana	1.35	721–910	29	2.0
Flax	1.38	700–1000	60–70	2.3
Jute	1.23	325–770	37–55	2.5
Pineapple	1.50	1020–1600	71	0.8
Sisal	1.20	460–810	41	3.4
Kenaf	1.20	745–930	41	1.6
Hemp	1.35	530–1100	45	3.0
Cotton	1.21	250–500	6–10	7.0

**Table 2:** List of obtained PU-based composites.

Filler	Filler Content [%]	Sample Designation
None/Pure PU matrix	-	PU
MCC	1	1% MCC/PU
MCC	2	2% MCC/PU
MCC	5	5% MCC/PU
MCC	10	10% MCC/PU
PALF	1	1% PALF/PU
PALF	2	2% PALF/PU
PALF	5	5% PALF/PU
PALF	10	10% PALF/PU
modPALF	1	1% modPALF/PU
modPALF	2	2% modPALF/PU
modPALF	5	5% modPALF/PU
modPALF	10	10% modPALF/PU

**Table 3:** Tensile strength test results of the created materials.

Sample Name	R_m_ [MPa]	E [MPa]	ε_max_ [mm] ^1^
PU	15.1 ± 1.0	140 ± 12	130 ± 28
1% MCC/PU	11.7 ± 2.4	156 ± 16	113 ± 31
2% MCC/PU	7.7 ± 1.9	180 ± 20	81 ± 14
5% MCC/PU	5.0 ± 0.2	308 ± 28	64.17 ± 10
10% MCC/PU	1.9 ± 0.7	597 ± 54	11 ± 4
1% PALF/PU	7.1 ± 2.9	130 ± 11	126 ± 68
2% PALF/PU	7.5 ± 0.3	149 ± 12	123 ± 29
5% PALF/PU	2.2 ± 0.1	251 ± 23	11 ± 2
10% PALF/PU	1.2 ± 0.3	503 ± 48	7 ± 4
1% modPALF/PU	9.3 ± 3.8	169 ± 13	113 ± 61
2% modPALF/PU	10.7 ± 1.4	195 ± 21	110 ± 26
5% modPALF/PU	3.9 ± 0.4	325 ± 29	10 ± 2
10% modPALF/PU	1.7 ± 0.1	710 ± 68	6 ± 3

^1^ initial sample length 150 mm.

**Table 4:** DSC results for PU and PU/MCC composites from the first heating run.

Sample Name	Melting Point of Soft Segments [°C]	Heat of Fusion First Cycle [J/g]	Degree of Crystallinity [%]	Heat of Fusion Second Cycle [J/g]	Degree of Crystallinity [%]
PU	17	28.90	19.27	-	-
1% MCC/PU	18	28.69	18.94	-	-
2% MCC/PU	17	29.58	19.33	-	-
5% MCC/PU	22	34.55	21.65	25.57	16.02
10% MCC/PU	24	31.36	19.01	25.09	15.20

**Table 5:** DSC results for PU and PU/PALF composites from the first heating run.

Sample Name	Melting Point [°C]	Heat of Fusion First Cycle [J/g]	Degree of Crystallinity [%]	Heat of Fusion Second Cycle [J/g]	Degree of Crystallinity [%]
PU	17	28.90	19.27	-	-
1% PALF/PU	19	27.84	18.37	2.37	1.56
2% PALF/PU	18	28.05	18.33	6.30	4.12
5% PALF/PU	20	26.95	16.89	26.65	16.70
	36	4.96	3.11	-	-
10% PALF/PU	19	24.05	14.58	26.49	16.05
	36	6.32	3.83	-	-

**Table 6:** DSC results for PU and PU/modPALF composites from the first heating run.

Sample Name	Melting Point [°C]	Heat of Fusion First Cycle [J/g]	Degree of Crystallinity [%]	Heat of Fusion Second Cycle [J/g]	Degree of Crystallinity [%]
PU	17	28.90	19.27	-	-
1% modPALF/PU	26	44.19	29.17	42.59	28.11
2% modPALF/PU	18	11.99	7.91	-	-
	39	37.74	24.67	34.28	22.40
5% modPALF/PU	18	13.50	8.82	-	-
	37	35.51	22.02	32.49	20.36
10% modPALF/PU	17	9.96	6.24	-	-
	39	47.75	28.94	31.89	19.33

**Table 7:** Thermal stability (TG) test results of polyurethane and its composites.

Sample Name	T_1%_ [°C]	T_2%_ [°C]	T_3%_ [°C]	T_5%_ [°C]	T_50%_ [°C]	Char Residue [%]
PU	280	295	303	313	397	0.15
1% MCC/PU	292	309	318	328	389	4.56
2% MCC/PU	293	309	317	327	390	4.69
5% MCC/PU	287	303	311	321	389	5.15
10% MCC/PU	286	305	312	323	394	5.78
1% PALF/PU	277	294	303	314	383	1.89
2% PALF/PU	241	273	287	300	380	2.56
5% PALF/PU	243	273	286	300	380	2.91
10% PALF/PU	217	256	273	292	384	3.42
1% modPALF/PU	249	268	281	297	388	4.17
2% modPALF/PU	266	290	303	317	395	5.76
5% modPALF/PU	235	267	285	307	398	6.62
10% modPALF/PU	243	270	291	307	398	8.34

**Table 8:** DTG analysis of PU and PU composites modified with MCC, PALF and modPALF.

Sample Name	T_maxDTG1_ [°C]	maxDTG1 [%/°C]	T_maxDTG2_ [°C]	maxDTG2 [%/°C]	T_maxDTG3_ [°C]	maxDTG3 [%/°C]
PU	361	0.53	408	2.66	-	-
1% MCC/PU	371	1.08	399	1.55	-	-
2% MCC/PU	354	0.84	402	1.37	-	-
5% MCC/PU	354	0.65	391	1.84	-	-
10% MCC/PU	351	0.50	395	1.78	-	-
1% PALF/PU	344	0.73	391	1.39	422	0.33
2% PALF/PU	323	0.45	387	1.24	417	0.45
5% PALF/PU	340	0.56	390	1.13	417	0.46
10% PALF/PU	322	0.42	390	1.06	418	0.47
1% modPALF/PU	339	0.50	398	1.27	-	-
2% modPALF/PU	355	0.57	403	1.58	-	-
5% modPALF/PU	356	0.46	403	1.42	-	-
10% modPALF/PU	356	0.48	404	1.56	-	-

## Data Availability

The original contributions presented in this study are included in the article. Further inquiries can be directed to the corresponding author.
